# Assessment of Histological Features in Squamous Cell Carcinoma Involving Head and Neck Skin and Mucosa

**DOI:** 10.3390/jcm10112343

**Published:** 2021-05-27

**Authors:** Ana Caruntu, Liliana Moraru, Mihai Lupu, Diana Alina Ciubotaru, Marius Dumitrescu, Lucian Eftimie, Radu Hertzog, Sabina Zurac, Constantin Caruntu, Oana Cristina Voinea

**Affiliations:** 1 Department of Oral and Maxillofacial Surgery, “Carol Davila” Central Military Emergency Hospital, 010825 Bucharest, Romania; ana.caruntu@gmail.com (A.C.); liliana.moraru@yahoo.com (L.M.); 2 Department of Oral and Maxillofacial Surgery, Faculty of Dental Medicine, “Titu Maiorescu” University, 031593 Bucharest, Romania; 3 Dermatology Research Laboratory, “Carol Davila” University of Medicine and Pharmacy, 050474 Bucharest, Romania; lupu.g.mihai@gmail.com; 4 Department of Physiology, “Carol Davila” University of Medicine and Pharmacy, 050474 Bucharest, Romania; diana-alina.ciubotaru@drd.umfcd.ro; 5 Department of Pathology, “Carol Davila” Central Military Emergency Hospital, 010825 Bucharest, Romania; marius_med@yahoo.com (M.D.); lucicaeftimie@yahoo.com (L.E.); 6 “Cantacuzino” National Medico-Military Institute for Research and Development, 050096 Bucharest, Romania; raduhg@yahoo.co.uk (R.H.); oanacristinavoinea@gmail.com (O.C.V.); 7 Department of Pathology, “Carol Davila” University of Medicine and Pharmacy, 050474 Bucharest, Romania; 8 Department of Pathology, Colentina University Hospital, 020125 Bucharest, Romania; 9 Department of Dermatology, “Prof. N.C. Paulescu” National Institute of Diabetes, Nutrition and Metabolic Diseases, 011233 Bucharest, Romania

**Keywords:** squamous cell carcinoma, head and neck, tumor/stroma ratio, immune infiltration, tumor budding, necrosis, histopathology, prognostic

## Abstract

Background: squamous cell carcinoma (SCC) is the second most common type of malignancy worldwide. Skin and mucosa of the head and neck areas are the most frequently affected. An aggressive behavior in SCC is not easily detected, and despite all efforts, mortality in these types of cancer did not show major improvements during recent decades. In this study, we aim to determine the role of histological features available through standard pathology assessment in SCC and their relation with tumor behavior and patients’ survival. Method: in a group of one hundred patients diagnosed with SCC involving the head and neck areas, we assessed the presence of four histological features (tumor/stroma ratio, immune infiltration at the front of invasion, tumor-budding activity, and tumor necrosis), their correlations with tumor type (mucosal or cutaneous), tumor clinicopathological characteristics, and their prognostic potential. Results: the comparison between histological features in cutaneous versus mucosal SCC reveals no significant differences for any of the four parameters assessed. We found significant correlations between tumor/stroma ratio and lymphatic metastasis (*p* = 0.0275), perineural invasion (*p* = 0.0006), and clinical staging (*p* = 0.0116). Immune infiltration at the front of invasion revealed similar correlations with lymph node involvement (*p* = 0.002), perineural invasion (*p* = 0.0138), and clinical staging (*p* = 0.0043). Tumor budding and tumor necrosis correlated with the size of the tumor (*p* = 0.0077 and *p* = 0.0004) and the clinical staging (*p* = 0.0039 and *p* = 0.0143). In addition, tumor budding was significantly correlated with perineural invasion (*p* = 0.0454). In mucosal SCC, patients with improved outcome revealed high values for the tumor/stroma ratio (*p* = 0.0159) and immune infiltration at the front of invasion (*p* = 0.0274). However, the multivariate analysis did not confirm their independent prognostic roles. Conclusions: extended histological assessments that include features such as tumor/stroma ratio, immune infiltration at the front of invasion, tumor budding, and tumor necrosis can be an easy, accessible method to collect additional information on tumor aggressiveness in skin and mucosa SCC affecting the head and neck areas.

## 1. Introduction

Squamous cell carcinoma (SCC) represents the second most common type of malignancy worldwide [1]. It arises from the squamous layer of the epithelium. The head and neck skin and the mucosal layers are responsible for most SCCs. More than 70% of cutaneous SCC emerges from head and neck areas [1], while head and neck cancers (HNC) developing from the mucosal layer of the aero-digestive tube are almost exclusively of the SCC type [2]. Despite major progress in developing new preventive, diagnostic, and therapeutic tools, the worldwide incidence of SCCs has increased continuously and the survival rates did not show major improvements in recent decades [3,4]. Patients affected by this devastating disease are often disabled and disfigured, either by disease progression or by therapeutic sequelae, with major challenges in social reintegration, increasing the public and economic burden associated with SCCs. Therefore, the interest in developing biomarkers with prognostic potential has increased for this type of malignancy [5,6,7,8,9], alongside other cancers [10,11,12]. Boundaries were crossed and researchers have identified sophisticated methods to stratify patients by risk or response to therapy in many cancers. However, most of these biomarkers require equipment, kits, or special training specific for a research lab that rarely can be implemented in a standard clinical setting [13,14]. Clinical and histopathological assessments are still the main prognostic tools routinely used in medical practice. In SCCs, histopathological features such as degree of differentiation, perineural invasion, and vascular invasion are widely used as prognostic elements in patient assessment [15,16]. However, histopathological investigation can provide much more valuable information regarding tumor cell architecture, stromal component, local immune response, and the presence of necrosis that might carry additional prognostic potential. These histopathological features have proven their predictive value in different types of cancers and are currently used in clinical practice [17,18,19,20].

In this study, we aim to assess the prognostic potential of four histopathological features available on standard hematoxylin-eosin (HE) staining, namely tumor/stroma ratio, local immune response, tumor-budding activity, and tumor necrosis, in SCCs involving distinct areas of the head and neck. In addition, we aim to identify any relevant differences in terms of histopathological behavior between the two major types of SCC, specifically oral SCC (OSCC), affecting the oral and lip mucosa, and cutaneous SCC (CSCC).

## 2. Materials and Methods

### 2.1. Patients Selection

Patients with histologically confirmed diagnosis of squamous cell carcinoma (SCC) were included in this study. All patients were treated in the Department of Oral and Maxillofacial Surgery, “Carol Davila” Central Military Emergency Hospital Bucharest. The study was conducted in accordance with ethical guidelines and received the approval of the local ethics committee (No. 25/27 November 2017). The inclusion criteria for our study were patients with confirmed diagnosis of cutaneous or mucosal SCC who did not receive any previous treatment and were eligible for curative surgery. Patients with unresectable or metastatic tumors, with incomplete medical records, or who were lost to follow-up were excluded. All patients underwent a thorough preoperative workup (clinical and imagistic assessment) followed by radical resection of the tumor. Neck dissection was performed in all patients with positive nodes as well as in patients with large tumors (T stages 3 and 4A) and clinically negative nodes. Surgery was followed by radiotherapy with or without chemotherapy in accordance with the national guidelines and entered a follow-up program, with visits that included clinical and imagistic assessments.

### 2.2. Histological Evaluation Criteria

**Figure 1:**
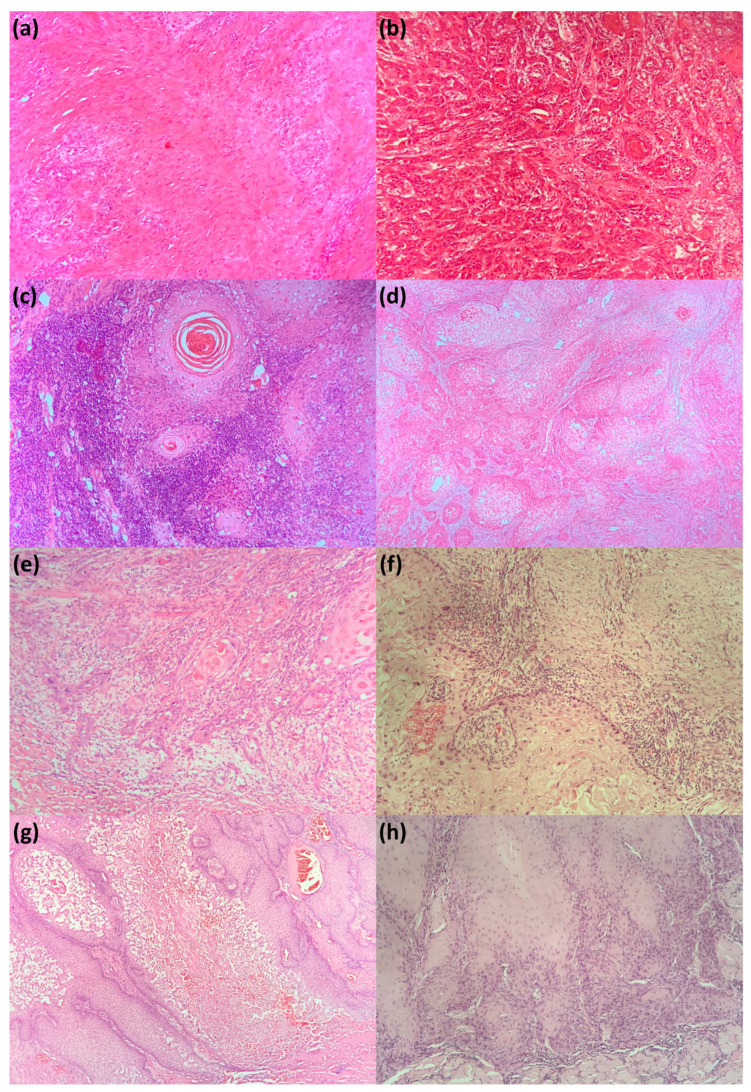
Histological features in SCC: (**a**) intense desmoplastic reaction (tumor/stroma ratio < 1) in non-keratinizing SCC; HE, original magnification 20×; (**b**) poor desmoplastic reaction (tumor/stroma ≥1) in moderately differentiated SCC; HE, original magnification 10×; (**c**) abundant immune infiltrate in keratinizing, well-differentiated SCC; HE, original magnification თ; (**d**) absence of tumor-infiltrating lymphocytes in moderately differentiated SCC, HE, and original magnification 4×; (**e**) high tumor-budding activity in moderately differentiated SCC; HE, original magnification 10×; (**f**) low tumor-budding activity in moderately differentiated SCC; HE, original magnification 10×; (**g**) extended necrotic areas in moderately differentiated SCC; HE, original magnification 20×; and (**h**) absence of necrotic areas in moderately differentiated SCC, keratinizing SCC; HE, original magnification 20×. SCC: Squamous cell carcinoma; HE: hematoxylin-eosin.

All specimens were fixed in formalin 10% and were cut according to American Joint Committee on Cancers (AJCC) protocol applied with the particularities of each case. After pathological orientation, tumor fragments and, where the case imposed, surgical margins and lymph nodes were embalmed in paraffin blokes, cut at 5 µm, and automatically stained in HE using the Leica histopathology system. Representative slides were selected for examination, and tumor samples with viable cells were evaluated, assessing four histological features: tumor/stroma ratio, immune infiltration at the front of invasion, tumor-budding activity, and tumor necrosis (see Figure 1). Each case was analyzed by two different experienced pathologists, and whenever there were divergent opinions, a third pathologist was involved.

For the selected cases, we assessed the ratio between tumor cells, individualized or clustered tumor cells with or without keratinization, and stromal response (fibrous, eosinophilic, and paucicellular areas) and quantified them according to the majority of the component as stromal poor (tumor/stroma ratio >1) or stromal rich (tumor/stroma ratio ≤1), as previously reported in other studies [21]. We assessed the presence of inflammatory infiltrate, regardless of its phenotype. As we found no standardization in the literature, we applied the method of evaluating tumor-infiltrating lymphocytes used in breast carcinoma [22]. We scanned the slides at low magnification, and after analyzing in detail the presence of lymphocytes, easily distinguishable in HE according to their dimension, shape, and lack of nuclear atypia, and comparing to tumor cells in the vicinity, we classified them into two variables, high or low, considering the cutoff to be an inflammatory infiltrate that surrounds the entire tumor bed, the presence of lymphocyte follicles, or a thick band of more than 5 rounds of inflammatory cells that surrounds at least 50% of the diameter of the tumor bed. Tumor budding was assessed in all tumor fronts, independently of their dimension, and we considered as cutoff point of up to 5 foci of tumor cells in the tumor stroma, stratifying patients into high and low tumor-budding activity based on the protocol reported for colorectal cancer [23]. For the tumor necrosis criteria, we considered a cutoff point ≤10% for classifying tumor as high or low in necrotic areas. After a complete assessment of the tumor bed, tumor necrosis areas with a histopathologic appearance of homogenous clusters of dead cells or cells organized into groups forming a coagulum containing cytoplasmic and nuclear debris, we quantified them as described in previous studies on other types of cancer [20] and classified them into one of the groups defined in our study.

### 2.3. Statistical Analysis

Statistical analysis was conducted with SPSS version 26 software package (IBM, Chicago, IL, USA) and Prism 9 software (GraphPad Software, San Diego, CA, USA). We used the Chi square test for the analysis of categorical data in order to identify potential relations between histological features and clinicopathological characteristics of the tumor. For the outcome correlations, we divided our study group in two categories: the first group included patients that were alive at the last follow-up call or patients that died due to other causes (OS), and the second group included patients that died due to disease progression (DRD). Furthermore, patient outcomes were analyzed by taking into consideration that mucosal and cutaneous SCC are two distinct entities in terms of etiopathogenesis and prognosis. Thus, for the outcome and survival analysis, we divided our study group into OSCC and CSCC. Survival curves were drawn using the Kaplan–Meier method, and the differences between groups were calculated with the Log-rank test. Multivariate analysis was carried out using Firth logistic regression, with the purpose of identifying significant clinical or pathological predictors concerning patient survival. For our model, we chose tumor to stroma ratio, inflammatory infiltrate, tumor-budding areas, and tumor necrosis as the four intended predictors. Statistical significance was considered for *p* < 0.05.

## 3. Results

### 3.1. Patients Characteristics

A demographic description of our patient group, and the clinical and histopathological characteristics of their tumors are presented in Table 1. From a total number of 147 patients with histologically confirmed SCC involving head and neck areas, 100 patients met the eligibility criteria and were included in this study. The mean age of the patients was 64.92 years old. Most of the patients were male, with a male–female ratio approaching 4:1. Fifty-seven patients were confirmed to have a history of long-term smoking, and forty-three reported a history of alcohol consumption. Long-term ultraviolet exposure (UV), habitual or professional, was confirmed in all patients. The primary site of the tumor was oral and lip mucosa for 79% of the patients, whereas tumors developing from the skin of the head and neck areas represented 21% of the cases. The mean follow-up interval was 37 months, with an overall survival range from 5 months to 61 months. Loco-regional recurrence was recorded in 29% of patients, and 23 patients died due to disease progression. Neck dissection, ipsilateral or bilateral in tumors involving the midline, was performed in 35 patients. Of these, 51% had histologically confirmed lymph node involvement, while the rest of the 17 patients (49%) had no regional dissemination of the disease. Histopathologic characteristics in our study group revealed that 79% of patients had a tumor/stroma ratio above 1. Tumor necrosis was seldom found in our patients, with only 16% of the patients displaying high rates of tumor necrosis. An analysis of the tumor immune response at the invasion front revealed that 67% of patients had high immune infiltration, whereas tumor-budding activity for the cutoff value of 5 was evenly distributed within the group. Thus, 48% of the patients showed high rates of tumor-budding activity, while 52% displayed no tumor budding or a maximum of five foci in tumor stroma.

### 3.2. Correlation Analysis between Histological Features and Clinicopathological Characteristics in SCC

The results of the correlation analysis between histopathological features of the tumors and their different clinicopathological characteristics are presented in Table 2. Tumor-budding activity and tumor necrosis were significantly correlated with tumor dimensions (*p* = 0.0077 and *p* = 0.0004, respectively). Regional lymph node dissemination revealed significant correlations with tumor/stroma ratio (*p* = 0.0275) and immune cell infiltration (*p* = 0.002). All histological parameters—tumor/stroma ratio, immune infiltration at the front of invasion, tumor-budding activity, and tumor necrosis—were significantly associated with clinical TNM staging in SCC (*p* = 0.0116, *p* = 0.0043, *p* = 0.0039, and *p* = 0.0143, respectively). Histologically confirmed perineural invasion was in strong association with tumor/stroma ratio, immune infiltration, and tumor-budding activity (*p* = 0.0006, *p* = 0.0138, and *p* = 0.0454, respectively). Tumor recurrence revealed significant correlations with the tumor/stroma ratio (*p* = 0.0026) and tumor budding (*p* = 0.0292) and approached the threshold of statistical significance for immune infiltration at the front of invasion (*p* = 0.0596). We found no correlations between histological features of the tumor and other clinicopathological characteristics such as gender, the degree of differentiation, and the keratinizing character of the tumor.

### 3.3. Assessment of Histological Features in Relation to SCC Type: OSCC Versus CSCC

The evaluation of correlations between the two major SCC types, CSCC and OSCC, and the histopathological features assessed in our study did not reveal any statistically significant differences. Thus, in both types of tumors, arising from mucosal keratinocytes or skin keratinocytes, we found all of the histological elements of interest in our study, with no particular pattern of distribution. The results are shown in Table 3.

### 3.4. Clinicopathological and Histological Correlations with Patient Outcome

For the patient outcome analysis, we divided our study group into the two types of SCC: cutaneous and mucosal, considering their distinct etiopathogenic and prognostic behaviors. For the CSCC, a valid outcome analysis could not be conducted due to the low number of patients that died due to disease progression (only two cases). In the OSCC group that included 79 patients, at the end of the follow-up period, 58 patients were alive and 21 have died secondary to disease progression. An analysis of correlations between the clinicopathological characteristics and patients’ outcome revealed that tumor size and TNM staging strongly correlated with survival (*p* = 0.0035 and *p* = 0.0001, respectively). Histologically confirmed regional lymph node dissemination presented a close association with poor prognosis in OSCC, without crossing the threshold for statistical significance (*p* = 0.0570). Exposure to known risk factors—tobacco and alcohol—was reported more frequently in patients with worse outcome (*p* = 0.0269 and *p* = 0.0092, respectively). Residual positive margins after tumor resection were associated with poor patient outcome (*p* = 0.0091).

Tumor histological characteristics in relation to patient outcome showed correlations for immune infiltration at the front of invasion and for tumor/stroma ratio. Thus, better survival rates were seen in patients that had a high tumor/stroma ratio, of more than 50% (*p* = 0.0159), and a high immune infiltration at the front of invasion (*p* = 0.0274). We found no statistically significant correlations with patient’s outcome for tumor-budding activity and for tumor necrosis (*p* = 0.1263 and *p* = 0.2854, respectively). The results are presented in Table 4.

### 3.5. Survival Analysis

**Figure 2:**
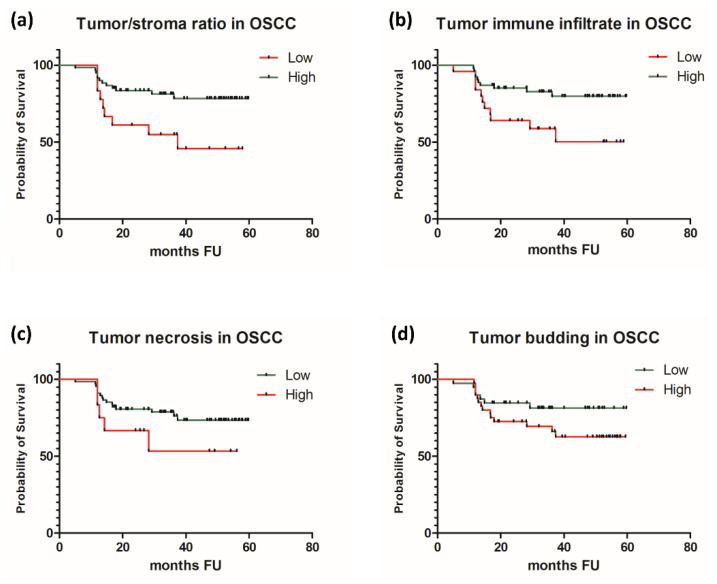
Survival curves in OSCC for (**a**) tumor/stroma ratio, (**b**) immune infiltration at the front of invasion, (**c**) tumor necrosis, and (**d**) tumor budding.

**Figure 3:**
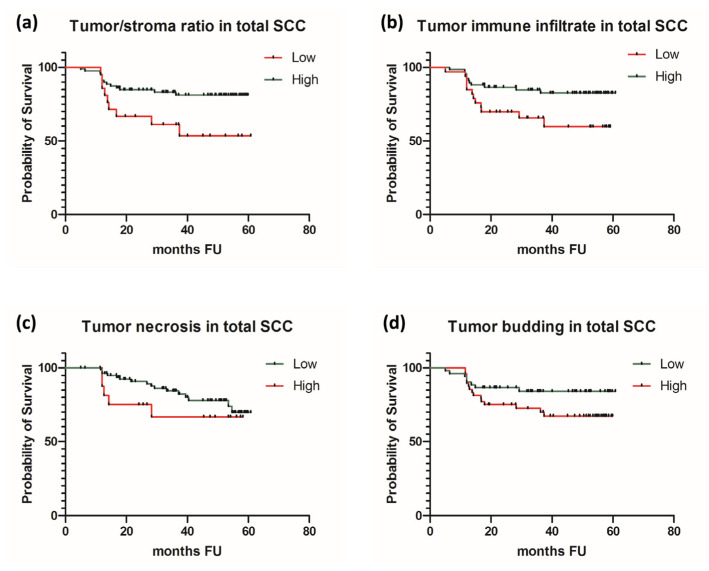
Survival curves in total SCC for (**a**) tumor/stroma ratio, (**b**) immune infiltration at the front of invasion, (**c**) tumor necrosis, and (**d**) tumor budding.

In order to overcome the limitations related to a reduced number of patients that died due to the progression of CSCC (only two patients), we conducted a survival analysis first on the subgroup of OSCC and afterwards of the total group of patients—OSCC and CSCC—looking for any differences that might be caused by the addition of a CSCC subgroup of patients. In OSCC, we found significant prognostic correlations for tumor/stroma ratio and immune infiltration at the front of invasion. Adverse prognostic factors were stroma reach tumors and immuno-deficient tumors (*p* = 0.0138 and *p* = 0.0164, respectively). In contrast, high tumor/stroma ratio and abundant immune infiltration at the front of invasion were associated with improved patients’ survival rates in OSCC. Tumor necrosis and tumor-budding activity did not reveal prognostic potential in our group of patients with OSCC. Survival analysis conducted on the entire group of patients, regardless of the type of SCC, revealed similar results, confirming the predictive potential for two out of four histological features: tumor/stroma ratio and tumor immune infiltration. Thus, stroma-rich tumors and immuno-deficient tumor at the front of invasion were negative prognostic elements in SCC, regardless of the mucosal or cutaneous subtype. As in OSCC, in the entire group of patients, tumor-budding activity and tumor necrosis were not correlated with patients’ survival (Table 5 and Figure 2 and Figure 3).

Multivariate analysis was run through penalized (Firth) logistic regression for both OSCC and total SCC. The model was significant at *p* = 0.04, and the results revealed that none of the four chosen pathological attributes showed significant prediction power for patient survival (the chance of a patient belonging to the OS or DRD groups): tumor to stroma ratio (*p* = 0.451 and *p* = 0.274, respectively), inflammatory infiltrate (*p* = 0.103 and *p* = 0.128, respectively), tumor-budding activity (*p* = 0.433 and *p* = 0.242, respectively), and tumor necrosis (*p* = 0.364 and *p* = 0.545, respectively).

## 4. Discussion

In the history of the fight against cancer, tumor cells captured initially all of the attention in scientific research. With time, it was shown that tumor cells together with the tumor microenvironment (TME) act as an independent entity with complex interactions that influence the process of carcinogenesis [24,25,26]. In our study, we assessed the clinicopathological correlations and prognostic capabilities of non-cellular and immune elements from TME alongside architectural disposal and viability of tumor cells in SCC affecting different head and neck areas. OSCC and CSCC are known as distinct entities in terms of ethiopathogenic and prognostic behavior; however, our result showed that the pathological characteristics of these two types of SCC are quite similar. In our study group, we found a proportional distribution of features such as tumor/stroma ratio, immune infiltration at the front of invasion, tumor budding, and tumor necrosis between OSCC and CSCC.

The exhaustive analysis that included all SCCs, regardless of type, revealed that the stromal component was strongly related to lymph node invasion, neural invasion, and clinical staging of the tumors. Our results showed that stroma-poor tumors were associated with a reduced incidence of lymph node metastasis and perineural invasion. Furthermore, in advanced clinical stages, stroma-rich tumors were found more frequently compared to incipient stages, where the stroma-poor character was almost exclusively seen. All of these findings suggest that an aggressive tumor behavior can be attributed to stroma-rich tumors in SCC. Survival analysis in relation to tumor/stroma ratio was consistent for both OSCC and total SCC and revealed a better prognosis for patients with stroma-poor tumors. Our findings are in accordance with results reported for many types of cancers, where tumor/stroma ratio is considered a prognostic factor. In solid tumors, such as breast carcinoma [27], epithelial ovarian cancer [28], non-small cell lung cancer [29,30], or colon cancer [31], this histopathological parameter is widely used in patients’ assessment. In SCC tumors, the tumor/stroma ratio was investigated for laryngeal and pharyngeal sites, where the authors have reported results similar to our findings [32]. In these subsites of SCC, stroma-rich tumors were found in patients with advanced disease stages and with lymph node metastasis and were associated with a poor response to chemotherapy and low survival rates. In our study group, one third of the OSCCs were of the stroma-rich type, while the rest were of stroma-poor character. Similar results were reported in another study that found that 30% of early staged tongue SCC tumors were of the stroma-rich type and that this characteristic was associated with poor patient outcome [33]. In CSCC, about 1 in 10 patients showed the presence of a stroma-rich pattern. An extensive study assessing the desmoplastic reaction in CSCC reported a similar distribution, with 8% of patients having stroma-rich tumors, that was associated with high incidences of local recurrence and metastasis, and poor patients outcome [34]. The desmoplastic reaction inside a tumor is the defensive response of the host against tumor invasion. It represents the proliferation of fibroblasts under several hypoxic triggers and contributes to biological changes that impact tumor response to neo-adjuvant treatment, including radio or chemotherapy resistance [35]. However, an intense desmoplastic reaction was found predominantly in patients with poor outcome compared to stroma-poor tumors that were associated with better survival rates. This might be explained by the aggressive character of the tumors that induces a more intense defensive reaction from the host with an increased desmoplastic response. Our results support this assumption, considering that the tumor/stroma ratio did not hold the independent prognostic character in multivariate analysis. Several studies reported similar results, concluding that a sub-unitary tumor/stroma ratio is highly suggestive for a poor prognosis [36]. To this date, for SCC, there is no consensus in the utility of evaluating tumor/stroma ratio and there are no guidelines to assess this histopathological characteristic as part of standard care. However, there is strong evidence to suggest that it can be an easy, accessible method to additionally stratify patients in risk groups.

Antitumor immune responses have been intensively studied during the last decades, with major breakthroughs in unveiling the mechanisms of carcinogenesis and, most importantly, the discovery of targeted immune therapies that revolutionized the outcomes in many types of cancers, making the impossible possible [37,38,39]. Both in cutaneous and mucosal SCCs, it has been shown that an abundant tumor infiltration with CD8^+^ lymphocytes was associated with improved outcome and better response to immunotherapy [3,40]. Similar findings were reported for CD56^+^ cells [41], whereas for CD4^+^ and CD20^+^ lymphocytes, there are contradictory results in terms of prognostic potential [42,43]. However, in order to retrieve all of this valuable prognostic information based on the types of immune cell tumor infiltration, special and sometimes expensive techniques are required that are not always available in a general clinical setting. In our study, we assessed the clinicopathological correlations and the predictive potential of the local antitumor immune response by using a non-discriminative, easy-to-apply qualitative measurement of immune cell infiltration at the front of invasion. We found that an intense immune infiltration at the front of invasion was associated with an improved patient outcome in both the OSCC subgroup and the total SCC group of patients. The overall analysis revealed that the immune infiltrate at the front of invasion was correlated with nodal status, perineural invasion, and TNM clinical staging. Aggressive SCC tumors with regional lymph node dissemination and neural invasion displayed a weak immune response on HE slides. In addition, early stage tumors were associated with an abundant immune infiltrate at the front of invasion, while in advanced stages, the number of tumors with a low immune reaction at the front of invasion increased dramatically. Tumor progression from initial stages to advanced disease is not entirely due to the intrinsic aggressiveness of malignant cells but is also facilitated by a progressively altered antitumor immune response that loses its capacity of tumor cell clearance, thus allowing for tumor growth and invasion [44]. Through the process of immunoediting, malignant cells develop protective mechanisms against immune recognition, enabling the immune escape, and induce an anergic status in immune cells that promotes tumor progression [45]. In both OSCC and CSCC, we found that two thirds of the tumors revealed a strong immunogenic character. It has been reported that tobacco and UV-associated malignancies, such as OSCC and CSCC, are characterized by the highest rates of DNA alterations that associate with an increased expression of tumor specific antigens and thus generate an intense immune response [46]. Better survival rates reported in CSCCs compared to other head and neck sites can be related to this strong immunogenic character in addition to other tumor characteristics, such as an easier early diagnosis and treatment due to the accessibility and visibility of the lesions [47]. Our findings are in accordance with the results reported in other studies that have identified significant correlations between tumor immune cells infiltration and clinicopathological tumor characteristics in different types of SCCs [48,49,50]. The analysis that we conducted provides the advantage of a simple, time-sparing method that requires no additional costs and training and can be easily implemented in the assessment of SCC tumors.

In our study group, we could not confirm the predictive potential enclosed in the analysis of tumor budding and tumor necrosis. The analysis of correlations with clinicopathological features in SCC revealed that both tumor budding and tumor necrosis related significantly with tumor dimensions and clinical staging. We noticed that, as the primary tumors grew larger, they were more frequently associated with a high tumor-budding activity. Advanced clinical stages were also associated with increased tumor-budding activity and extended tumor necrosis. In addition, perineural invasion was more frequently found in tumors with increased tumor-budding activity. Considering that locoregional recurrences were associated with an intense tumor-budding activity, this histologic element might be interpreted as a marker for an aggressive behavior in SCC tumors, even though in our study its prognostic character was not confirmed. In several carcinomas, especially in colorectal cancer, a high tumor-budding activity was correlated with poor prognosis and early metastatic events [23]. A recent meta-analysis reported results that support the predictive potential of tumor budding for oral SCC tumors; however there is no consensus on the cutoff point, with the interval ranging from 3 to 10 foci [51]. In colorectal cancer, tumor budding is defined as the presence of clusters of up to five tumor cells at the front of invasion that imply an aggressive tumor behavior. Tumor budding is seen as the expression of epithelial-to-mezenchymal transition (EMT), a characteristic of the colorectal tumor cells that gain increased plasticity features with altered intercellular adhesion, allowing for vascular invasion and distant metastasis, through circulating tumor cells (CTCs) [23]. Tumor cells in SCCs have been reported to have this ability to go through the process of EMT, experimentally confirmed by the presence of CTCs [52]. The presence of CTCs in head and neck SCC patients was associated with short disease-free survival rates, thus confirming the aggressive character of the tumor [53]. The tumor budding activity can be a simple, accessible alternative that can be used in histopathological tumor evaluation, providing additional information on the aggressiveness of SCC tumors.

Tumor necrosis is commonly seen in aggressive cancers. The main triggers for cell necrosis are ischemia and hypoxia, which can be often found in large tumors. Necrosis is generated by tumor overgrowth that exceeds the rhythm of neovascularisation [54]. In our study group, we found an increased incidence of extended necrotic areas, affecting more than 10% of the lesion, in large T4a tumors, where almost half of the lesions met the criteria for high necrotic activity, and in advanced stages of the disease, with 30% of tumors in stage IVA displaying high tumor necrosis. Experimentally induced death of the tumor cells was one of the therapeutic pathways explored in the long history of the fight against cancer. In most malignancies, tumor cell death is induced mainly through apoptosis [55]. However, epithelial cancers, including SCC, are known to be resistant to apoptosis, and tumor necrosis is commonly seen during tumor growth to advanced disease and as the result of many antitumor therapies [56]. Tumor necrosis is also associated with a status of chronic inflammation that can contribute to the aggressive tumor behavior [57]. Thus, the complex effects of the necrotic activity within tumor tissue is difficult to control, with tumor necrosis working as a dual element in cancer pathogenesis.

Alongside recent discoveries in the diagnosis of SCC, such as in vivo confocal microscopy that has proven its value in both cutaneous and mucosal SCC [58,59,60], allowing for early detection of malignant transformation, our findings can contribute with valuable information regarding a potentially aggressive tumor behavior, thus allowing for an optimal selection of therapeutic and a positive influence on the patients’ outcome.

## 5. Conclusions

Our study provides insight on the connections between certain histological characteristics easily identifiable though standard histopathological assessment—tumor/stroma ratio, tumor immune infiltration, tumor budding, and tumor necrosis—and the clinicopathological features of SCCs involving head and neck areas, including their prognostic potential. Each of the four analyzed histopathological features revealed significant correlations with different tumor characteristics, such as the size of the primary tumor, lymph node metastasis, perineural involvement, or clinical staging, all related to an aggressive behavior in SCC. Thus, this is an easy, cost effective method, accessible in any standard pathology setting that allows for the collection of additional histopathological information regarding tumor aggressiveness in SCC involving head and neck areas.

## Figures and Tables

**Table 1:** Descriptive statistics in Squamous cell carcinoma (SCC).

Variable		No (%)
Age (Mean ± SD), range, years	64.92 ± 12.78 (28–92)	
Gender		
	Male	73%
	Female	27%
Smoking		
	Smokers	57%
	Nonsmokers	39%
	Missing	4%
Alcohol consumption		
	Drinkers	43%
	Nondrinkers	53%
	Missing	4%
T stage		
	T1	28%
	T2	40%
	T3	16%
	T4	16%
Nodal status *		
	pN0	17%
	pN+	18%
TNM stage		
	I	27%
	II	33%
	III	14%
	IVA	26%
Location		
	Oral SCC	79%
	Cutaneous SCC	21%
Histological differentiation		
	High	26%
	Intermediate	55%
	Low	19%
Perineural invasion		
	Confirmed	10%
	Not confirmed	90%
Resection margins		
	Positive	11%
	Negative	89%
Locoregional recurrence		
	Present	29%
	Absent	71%
Tumor/stroma ratio		
	Low (≤1)	21%
	High (>1)	79%
Immune infiltrate		
	Low	33%
	High	67%
Tumor budding		
	Low (≤5)	52%
	High (>5)	48%
Necrosis		
	Low (≤10%)	84%
	High (>10%)	16%

* Neck dissection was performed for 35 patients.

**Table 2:** Correlation analysis between histological features and clinicopathological characteristics in SCC.

Variable	Tumor Stroma Ratio			Tumor Immune Response			Tumor Budding			Tumor Necrosis		
	≤50%	>50%	*p*	Low	High	*p*	Low	High	*p*	High	Low	*p*
Gender			0.999			0.999			0.822			0.999
Male	15	58		24	49		37	36		12	61	
Female	6	21		9	18		15	12		4	23	
T stage			0.1358			0.0722			0.0077			0.0004
T1	5	23		8	20		22	6		1	27	
T2	5	35		9	31		18	22		5	35	
T3	5	11		7	9		7	9		1	12	
T4A	6	10		9	7		5	11		9	10	
Nodal status (*n* = 35) *			*0.0275*			*0.002*			0.3175			0.1774
pN0	2	15		3	14		10	7		1	16	
pN+	9	9		13	5		7	11		5	13	
TNM stage			*0.0116*			*0.0043*			*0.0039*			*0.0143*
I	3	24		7	20		22	5		1	26	
II	3	30		5	28		15	18		5	28	
III	5	9		6	8		5	9		1	13	
IVA	10	16		15	11		10	16		9	17	
Histological differentiation			0.2211			0.2306			0.328			0.7481
High	3	23		6	20		15	11		3	23	
Intermediate	15	40		18	37		25	30		10	45	
Low	3	16		9	10		12	7		3	16	
Keratinization status			0.9319			0.6147			0.2909			0.708
Keratinizing	15	59		24	50		36	38		13	61	
Non-keratinizing	3	11		6	8		10	4		2	12	
Unknown	3	9		3	9		6	6		1	11	
Perineural invasion			*0.0006*			*0.0138*			*0.0454*			0.999
Confirmed	7	3		7	3		2	8		1	9	
Not confirmed	14	76		26	64		50	40		15	75	
Locoregional reccurence			*0.0026*			0.0596			*0.0292*			0.5481
Present	12	17		14	15		10	19		6	23	
Absent	9	62		19	52		42	29		10	61	

* From a total of 35 neck dissections; statistical significance *p* < 0.05.

**Table 3:** Assessment of histological features in relation to SCC type.

Histological Features		OSCC	CSCC	*p* Value
Tumor stroma ratio				0.5508
	Low	18	3	
	High	62	17	
Tumor immune infiltrate				0.6077
	Low	25	8	
	High	54	13	
Tumor budding				0.3364
	Low	39	13	
	High	40	8	
Tumor necrosis				0.7392
	High	12	4	
	Low	67	17	

Statistical significance *p* < 0.05.

**Table 4:** Correlation analysis between histological features, clinicopathological characteristics, and outcome in OSCC.

Variable		No	Survivors	Deceased	*p*
Tumor/stroma ratio					*0.0159*
	Low (≤50%)	18	50%	50%	
	High (>50%)	61	80%	20%	
Immune infiltrate					*0.0274*
	Low	25	56%	44%	
	High	54	81%	19%	
Tumor budding					0.1263
	Low (≤5)	39	82%	18%	
	High (>5)	40	65%	35%	
Tumor necrosis					0.2854
	Low (≤10%)	67	76%	24%	
	High (>10%)	12	58%	42%	
Sex					0.1305
	Male	61	69%	31%	
	Female	18	89%	11%	
Smoking					*0.0269*
	Smokers	53	64%	36%	
	Nonsmokers	22	91%	9%	
	Missing	4	100%	0%	
Alcohol consumption					*0.0092*
	Drinkers	38	58%	42%	
	Nondrinkers	37	86%	14%	
	Missing	4	100%	0%	
T stage					*0.0035*
	T1	16	94%	6%	
	T2	36	78%	22%	
	T3	15	73%	27%	
	T4	12	33%	67%	
Nodal status *					0.0570
	pN0	17	88%	12%	
	pN+	16	56%	44%	
TNM stage					*0.0001*
	I	15	100%	0%	
	II	29	90%	10%	
	III	13	62%	38%	
	IVA	22	41%	59%	
Histological differentiation					0.1116
	High	17	88%	12%	
	Intermediate	47	74%	26%	
	Low	16	56%	44%	
Perineural invasion					0.4430
	Confirmed	10	60%	40%	
	Not confirmed	69	75%	25%	
Resection margins					*0.0091*
	Positive	9	33%	67%	
	Negative	70	79%	21%	
Locoregional recurrence					*<0.0001*
	Present	27	22%	78%	
	Absent	52	100%	0%	

* Neck dissection was performed for 35 patients; statistical significance *p* < 0.05.

**Table 5:** Survival analysis.

Variable	Parameter	OSCC	Total SCC (OSCC and CSCC)
Tumor/stroma ratio	Log-rank test	6.064	5.588
	95% CI logrank	1.311 to 10.86	1.238 to 9.802
	*p* value	0.0138	0.0181
Immune infiltration	Log-rank test	5.76	4.495
	95% CI logrank	1.238 to 8.321	1.143 to 6.817
	*p* value	0.0164	0.034
Tumor budding	Log-rank test	1.926	2.707
	95% CI logrank	0.1160 to 1.445	0.2201 to 1.141
	*p* value	0.1433	0.0999
Tumor necrosis	Log-rank test	2.142	1.087
	95% CI logrank	0.2217 to 1.244	0.1631 to 1.741
	*p* value	0.1652	0.2972

Statistical significance *p* < 0.05.

## Data Availability

The datasets used and/or analyzed during the present study are available from the corresponding author.
